# The Incidental Diagnosis of a Hodgkin Lymphoma Following Elective Robotic-Assisted Inguinal Hernia Repair: A Case Report

**DOI:** 10.7759/cureus.92845

**Published:** 2025-09-21

**Authors:** Surkhaba Khan, Katherina Boettge, Juaquito Jorge

**Affiliations:** 1 Surgery, Saint James School of Medicine, Chicago, USA; 2 General Surgery, Franciscan Health, Olympia Fields, USA; 3 General and Bariatric Surgery, Tiesenga Surgical Associates, Elmwood Park, USA; 4 Surgery, Piedmont Weight Management and Bariatric Center, Augusta, USA

**Keywords:** hodgkin lymphoma, incidental cancer, inguinal hernia, medical education, robotic-assisted surgery

## Abstract

Hodgkin lymphoma (HL) is a rare malignancy, typically diagnosed via investigations prompted by clinical symptoms or imaging findings. In contrast, inguinal hernia repair, one of the most common elective surgeries, is rarely associated with malignancy. This case report describes an incidental discovery of stage IIIA HL during routine robotic-assisted laparoscopic hernia repair, underscoring the importance of thoroughly evaluating atypical intraoperative observations, as early recognition enables timely diagnosis and intervention. A 46-year-old male underwent elective robotic-assisted laparoscopic repair of a left inguinal hernia. Intraoperatively, an enlarged femoral mass, later identified as a retroperitoneal lymph node, was resected. Pathology confirmed stage IIIA HL. The patient received six cycles of chemotherapy and has since shown no recurrence during follow-up.

HL accounts for approximately 10% of all lymphomas, with several histological subtypes. Although hernia repairs are routine, incidental malignancies are rare. This report reinforces the need for vigilance and histopathological assessment of atypical findings, even during standard procedures. Early detection facilitates timely treatment and improves survival. Incidental detection of malignancy during routine surgery can significantly impact patient outcomes. This report demonstrates the importance of meticulous intraoperative assessment, enabling early intervention, improved survival, and reduced recurrence risk. Documenting such findings is essential to inform surgical best practices.

## Introduction

Hodgkin lymphoma (HL) is a neoplastic proliferation of lymphoid cells forming solid masses in lymph nodes or extranodal tissue [1]. It is characterized by Reed-Sternberg cells, large B cells with multilobed nuclei and prominent nucleoli (‘owl-eyed nuclei’), which are typically CD15- and CD30-positive [1]. Its clinical presentation varies, but approximately 25% of patients develop systemic symptoms such as fever, night sweats, and weight loss before detectable lymphadenopathy, potentially delaying diagnosis and disease management [2]. HL follows a bimodal age distribution, peaking in the early 20s and mid-60s [2]. While its exact cause remains unknown, the risk factors include genetic predisposition, Epstein-Barr virus (EBV) infection, and immunodeficiency states [3]. Environmental factors, such as pesticide exposure and certain geographical regions (e.g., Southern Asia, parts of Latin America), have also been implicated [3]. The five-year survival rate is approximately 89% in the United States and 85% globally [4].

## Case presentation

A 46-year-old male with a medical history of hypertension, hyperlipidemia, asthma, and a past surgical history of laparoscopic appendectomy, presented to the office for an evaluation of a left groin bulge, associated with increasing discomfort over the past several months. He denied any other associated symptoms. Routine preoperative laboratory workup did not show any abnormal findings, and a diagnosis of an uncomplicated, non-strangulated left inguinal hernia was made clinically.

During the robotic-assisted laparoscopic left inguinal hernia repair, initial trocar placement revealed no concerning findings. The development of the peritoneal flap proceeded uneventfully. However, during the dissection of the hernia sac, extensive fibrosis was noted between the sac and the vas deferens. A mass-like structure, resembling an enlarged retroperitoneal lymph node, was identified behind the cord structures in the femoral space. It was carefully dissected and sent for pathological evaluation. 

**Figure 1:**
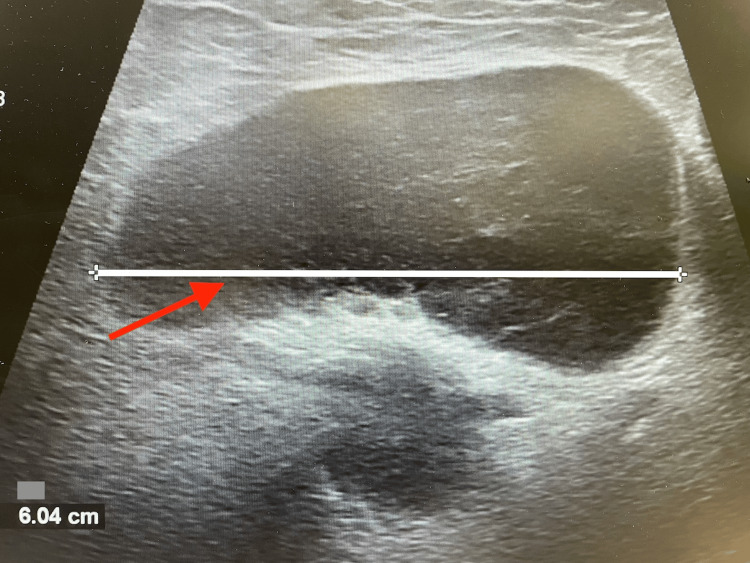
Ultrasound image of an enlarged inguinal lymph node measuring 6.04 cm in diameter

**Figure 2:**
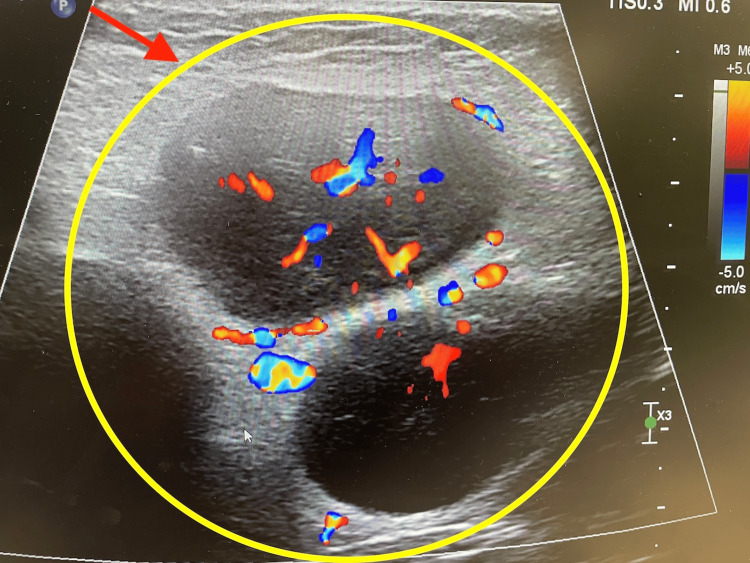
Ultrasound with color Doppler imaging demonstrating increased vascular flow

Postoperatively, a Doppler ultrasound of the inguinal lymph nodes was completed. In healthy adults, normal inguinal lymph nodes typically do not exceed 1.5 cm in diameter [5]. However, ultrasound imaging in this patient revealed multiple, significantly enlarged lymph nodes, including one inguinal lymph node measuring 6.04 cm (Figure 1). Additionally, color Doppler ultrasound demonstrated an increased vascular pattern (Figure 2). The combination of lymph node enlargement and increased blood flow raised concern for malignancy, necessitating further evaluation [6].

Initial pathology findings and final diagnosis confirmed stage IIIA classical HL, lymphocyte-rich, EBV-positive. The results were discussed with the patient, and he was referred to medical oncology. The patient was subsequently started on chemotherapy with BvAVD (brentuximab vedotin, doxorubicin, vinblastine, and dacarbazine) for a total of six cycles. HE underwent a PET-CT scan after two cycles, which showed resolution of all lymphadenopathies. He completed chemotherapy in April 2024 and underwent a follow-up PET-CT scan in June 2024, which showed no further evidence of malignancy.

## Discussion

Lymphomas are classified into Hodgkin lymphoma (HL) and non-Hodgkin lymphoma (NHL), with HL accounting for about 10% of cases [7]. The main subtypes include nodular sclerosis HL (NSHL), mixed cellularity HL (MCHL), lymphocyte-rich HL (LRHL), and lymphocyte-depleted HL (LDHL) [1]. NSHL is the most common variant, often exhibiting fibrosis and collagen band formation. MCHL, strongly associated with EBV infection, accounts for 40-50% of classical HL cases. LRHL has the most favorable prognosis, whereas LDHL, the most aggressive subtype, is more common in elderly and HIV-positive patients [1,2]. HL is staged using the Ann Arbor Classification (Table 1) [1,2,8].

**Table 1:** Ann Arbor Classification used for staging Hodgkin lymphoma

Stage	Description
Stage I	Stage involves a single lymph node region (I) or single extralymphatic site (Ie)
Stage II	Two or more lymph node regions on the same side of the diaphragm (II) or one lymph node region with a contiguous extralymphatic site (IIe)
Stage III	Lymph node involvement on both sides of the diaphragm, possibly including the spleen (IIIs) or limited extranodal involvement (IIIe, IIIes)
Stage IV	Disseminated involvement of one or more extralymphatic organs

Additionally, the following designations apply to any stage: the absence of systemic symptoms is indicated by “A”, the presence of systemic symptoms is indicated by “B”, and bulky disease, defined as a tumor ≥10 cm or >1/3 of the thoracic diameter, is indicated by “X” [1,2,8].

Treatment strategies depend on stage and risk factors. Standard therapies include chemotherapy, radiation therapy, stem-cell transplantation, and targeted immunotherapy for refractory or relapsed cases [3,9]. Current approaches aim to maximize survival while minimizing long-term toxicities such as secondary malignancies (e.g., leukemia, breast cancer after radiation) and cardiac damage [9]. In our patient’s case, the incidental discovery of stage IIIA HL during elective inguinal surgery underscores the critical need for vigilant evaluation in routine procedures. Primary or metastatic tumors within hernia sacs are exceedingly rare (<0.5%) [10]. With metastatic cases being even less common, a study of over 22,000 inguinal hernia repairs at the Mayo Clinic reported metastasis in only 0.07% of cases [10].

By contrast, the most frequent incidental finding during inguinal hernia repair is the presence of an occult contralateral hernia not detected on preoperative examination [11]. In a retrospective review of 297 patients undergoing robotic inguinal hernia repair, 15.8% were found to have an incidental contralateral hernia, suggesting the true incidence may be higher than previously appreciated [11]. Incidental intraoperative findings, whether benign or malignant, are clinically valuable. Although such findings may alter the course of treatment, they often improve patient outcomes by enabling earlier detection and timely management. At present, there are no studies or formal recommendations specifically addressing their management. Further research is required to establish standardized protocols for these uncommon but potentially significant occurrences.

The literature highlights variability in the decision to submit hernia sac specimens for histopathological analysis [10]. While routine submission may increase the likelihood of detecting malignancy, it also raises questions regarding cost-effectiveness [10]. Although the necessity of this practice remains uncertain, selective histopathological analysis in cases with suspicious features is prudent.

## Conclusions

We described a case of a 46-year-old male who underwent elective robotic-assisted laparoscopic inguinal hernia repair and was found to have an enlarged retroperitoneal lymph node, diagnosed as stage IIIA HL, lymphocyte-rich, EBV-positive. This report highlights the importance of meticulous macroscopic and microscopic examination during routine surgical procedures, especially when unusual findings are encountered. While the need for histopathological evaluation of all hernia sac masses remains a matter of debate due to the rarity of malignancy, selective examination in cases with atypical intraoperative findings can enable early diagnosis, significantly improving survival rates. Additionally, early detection facilitates robust treatments, reduces recurrence risk, and enhances outcomes for the patient. Furthermore, documenting such cases is crucial for improving patient outcomes and advancing medical knowledge. This report provides valuable data and contributes to the implementation of best practices for managing similar cases.
